# From Lab to Real World: Assessing the Effectiveness of Human Activity Recognition and Optimization through Personalization

**DOI:** 10.3390/s23104606

**Published:** 2023-05-09

**Authors:** Marija Stojchevska, Mathias De Brouwer, Martijn Courteaux, Femke Ongenae, Sofie Van Hoecke

**Affiliations:** IDLab, Ghent University-imec, Technologiepark-Zwijnaarde 82, 9052 Gent, Belgium

**Keywords:** human activity recognition, HAR, transfer learning, personalization, real-world data, convolutional neural networks

## Abstract

Human activity recognition (HAR) algorithms today are designed and evaluated on data collected in controlled settings, providing limited insights into their performance in real-world situations with noisy and missing sensor data and natural human activities. We present a real-world HAR open dataset compiled from a wristband equipped with a triaxial accelerometer. During data collection, participants had autonomy in their daily life activities, and the process remained unobserved and uncontrolled. A general convolutional neural network model was trained on this dataset, achieving a mean balanced accuracy (MBA) of 80%. Personalizing the general model through transfer learning can yield comparable and even superior results using fewer data, with the MBA improving to 85%. To emphasize the issue of insufficient real-world training data, we conducted training of the model using the public MHEALTH dataset, resulting in 100% MBA. However, upon evaluating the MHEALTH-trained model on our real-world dataset, the MBA drops to 62%. After personalizing the model with real-world data, an improvement of 17% in the MBA is achieved. This paper showcases the potential of transfer learning to make HAR models trained in different contexts (lab vs. real-world) and on different participants perform well for new individuals with limited real-world labeled data available.

## 1. Introduction

Human activity recognition (HAR) is the task of automatically recognizing human activities from signals collected from different devices, such as wearable sensors, sensors embedded in the environment, or vision devices [1]. Environment-sensor-based approaches are those where sensors are installed in objects that a person uses or interacts with. Examples of these can be found in smart homes equipped with sensors, such as light switch sensors and motion sensors and sensors to detect the power usage of appliances, light intensity, ${\mathrm{CO}}_{2}$, and humidity. In video-based approaches, movements are captured by a camera, and then computer vision techniques are applied to recognize the activities. One of the main concerns about these approaches is user privacy, and they are perceived as intrusive by users. Moreover, environment- and video-sensor-based approaches have the limitation of being able to infer only the activities within the area in which they are installed. This is incompatible with the life-style of the active population, whose daily locations are diverse, including: home, work, school, car, park, cafe, theater, cinema, supermarket, etc. While environmental devices can be useful for monitoring the elderly population, who spend most of the time at home [2,3], wearable devices, such as smartphones, chest- and wristbands, and body adhesive sensors, are more suitable for the active population. These devices are unobtrusive, comfortable, and equipped with motion sensors such as accelerometers and gyroscopes, which tend to have low power consumption. As such, they are a better fit for longitudinal and real-world activity monitoring across different locations. HAR based on accelerometers and/or gyroscopes has a broad range of applications, such as fitness tracking, sports, and healthcare.

Commercial wearable activity trackers have improved throughout the years, from only counting steps to detecting certain activities. The list of automatically detected activities is rather limited and usually (outdoor) sports-related (running, cycling). For most, it also takes up to several minutes before they detect the activity.

Since the beginnings of this research field, different methodologies and machine learning techniques, both traditional methods, such as support vector machines and boosted trees [4,5], as well as deep learning [6,7,8], have been proposed. In addition, a wide range of activities has been considered: low-level activities, such as sitting and running [9], daily activities, such as cooking and eating [10], and even hand gestures, such as sign language [11,12,13]. However, the vast majority of the researched methodologies have been evaluated on data that have been collected in laboratory settings. During data collection, participants have been asked to perform the activities for a certain amount of time, and they have been monitored during data collection [14,15,16]. This way, the execution of the activity is uninterrupted, and the start and end times of the execution are known with high precision. These HAR systems may perform poorly in real-world (RW) applications due to low generalization capabilities: each new user’s environment is different from the one used for data collection, their way of performing the activity is different, the activities are interrupted and interleaved, they place the wearable in different places, etc. Even though there has been a lot of investigation into overcoming the challenges of personal characteristics and the individual ways of performing activities by means of model personalization techniques, the majority has been evaluated on laboratory-collected data [17,18,19,20].

In this paper, we make the step towards building a HAR system for RW scenarios. To that end, we cover the following:

- We discuss the lack of research performed and evaluated on RW data and argue why the community should make the shift towards RW scenarios. We acknowledge and discuss the lack of publicly available RW data for activity recognition.
- To bridge the gap, we designed and performed longitudinal, unobserved RW HAR data collection from wrist-worn devices. We discuss challenges, such as recall bias and label quality, that are part of RW data collection and the steps we have taken to overcome these challenges. As a result, we have collected diverse accelerometer sensor data from 18 participants with different activities. This dataset fully reflects RW scenarios in the daily life of people.
- We trained and evaluated general models on this RW dataset, using different amounts of data. We discuss the amount of data needed to achieve satisfying results. We additionally identify challenges such as different approaches to labeling between subjects, (unintentional) mislabeling, and unknown watch placement (dominant vs. non-dominant arm).
- We investigated the potential of personalization on RW data and discuss whether and when it is of great value to perform personalization.
- We evaluated the predictive performance of models trained with data collected in a controlled environment on RW data and vice versa. We show that models trained on data in a controlled environment perform poorly when evaluated on RW data.
- We investigated the potential of personalizing models trained on data collected in controlled environments with RW data and show that their performance can be greatly improved with only few RW data.

The paper is structured as follows: in Section 2, we summarize the related work and discuss personalization and HAR in RW scenarios; in Section 4, we describe our data collection, proposed general models, and personalization method; in Section 5, we describe the different experiments we performed and present our results. In Section 6, we discuss certain aspects of our methodology and findings. Finally, in Section 7, we draw conclusions and discuss future work.

## 2. Related Work

Human activities can be classified into several levels and categories depending on the specific goal of the research or application. In the literature, the following prevalent classifications can be noted:

- Low-level activities: sitting, standing, lying down, walking, running, cycling, walking up/down the stairs, etc. [9].
- Activities of daily living (ADL): cooking, eating, sleeping, watching TV, reading a book, driving a car, shopping, etc. [10].
- Arm/hand gestures: hand waving, hand numbering, sign language, etc. [11,12,13].
- Sport-related activities: kicking a ball, throwing a ball, jumping, swimming styles, etc. [21,22].

In this section, we discuss existing research on HAR. We first take a deeper look into machine learning techniques adopted for HAR and continue by looking into the personalization of these HAR models. The section finishes by exploring the existing work and datasets on real-life data and scenarios, including a small discussion on the shortcomings of current approaches and highlighting the added value of our research.

### 2.1. HAR Machine Learning Techniques

In past research, HAR mostly consisted of engineering features from sensor signals. Usually the signal is first segmented using a (non-)overlapping fixed-size window (usually several seconds long). Each window is summarized by extracting statistical features in both the time and frequency domains, and represents a single sample. In this approach, researchers usually opt for the traditional machine learning (ML) techniques, such as k-nearest neighbors (kNN) [4,5], random forests (RF) [4,23], support vector machines (SVM) [4,5], and gradient-boosted trees (GBT) [5]. In the last years, however, (artificial) neural networks ((A)NN) and deep learning (DL) have found their applications in HAR as well, without requiring the need for feature extraction, for example: 1D and 2D convolutional neural networks (CNNs) [6,7,8], recurrent neural networks (RNNs) [24], and long short-term memory (LSTMs) [9,25]. Deep learning methodologies have the advantage of automatically learning features, but they require more labeled data than the traditional techniques [26,27].

### 2.2. Har Personalization of Models

The variability in data from different users influences the performance of the classifier when applied to data from a new user. This situation is very realistic: models are trained on pre-collected data and then deployed for monitoring the activity behavior of new users, depending on the specific application. As people differ in age, height, weight, and activity performance, the data from new users may be different from the data in the initial dataset, and the performance of the model might not meet the expected accuracy. One option is to first collect data from the new user and train a personal model for this person. This model would have low generalizing capabilities and might perform poorly if the person executed the activities somewhat differently than in the training data, e.g., walking slower. A lot of individual data would have to be collected and labeled in order to avoid overfitting. However, this is a long and cumbersome task.

A potential solution to this problem is the personalization of the general model. The idea is to initially train a general model from all the available data across multiple people, and then adapt that model using only a small amount of data from the specific individual for whom we want to deploy the model. Starting from a model that generalizes well, we overcome the challenge of overfitting on this user-specific data, yet improve the performance by additionally learning user-specific characteristics. In the literature, three main techniques for achieving this personalization of already existing HAR models can be discerned, i.e., incremental learning, domain adaptation, and transfer learning. Each one is briefly discussed below. There also exists research on personalizing HAR models based on similarity between users. We discuss this at the end of this section.

Several studies using incremental learning techniques have been published. Incremental learning is a method in which new incoming data are used to continuously update or adapt the existing model. Mannini and Intille [17] personalized an SVM model by adapting it to the new data. They used the algorithm presented in the work of Matic N. [28]. In this approach, a new SVM was trained on a set consisting of the support vectors from the original SVM augmented with newly arrived samples. Siirtola [18] used the Learn++ algorithm by adding new base models to an ensemble that was trained on user-specific data. They tested three base classifiers (linear discriminant analysis (LDA), quadratic discriminant analysis (QDA), and the classification and regression tree (CART)). Siirtola [29] later developed a context-aware method for personalization that could handle situations in which the body position of the sensor is not fixed. Although these studies observed improvements in accuracy after personalization, they were evaluated on data collected in controlled environments.

Next to incremental learning, another way to go about personalization is by domain adaptation. The goal of domain adaptation is to align the feature distribution of the target user with the rest of the users so that the model performs well on the target user. Mazankiewicz [19] combined both incremental learning and domain adaptation by using batch normalization for domain adaptation. During training, the data are split into batches, where each batch contains data from a single user and each batch is normalized with the statistics of the corresponding user. In this way, the distribution of the output of the fully connected layers is independent of the user. During the incremental learning part, the statistics for the unseen user are unknown and are initialized as the average of all previous users. As data come in, the statistics are updated, and they adapt to the target user. This brings the distribution of the target closer to the distribution of the source. They found that their method worked well for some but not all individuals. For some, the accuracy even decreases. This work is also evaluated on data collected in a controlled environment.

With the great success of NN (neural networks) in HAR, transfer learning using NN has also found its application in the problem of the personalization of HAR models. Seyed Ali Rokni and Marjan Nourollahi [20] used CNNs to transfer knowledge learned from the source group of people to the target user. They achieved this by freezing all layers except the classification layer. They then adjust the weights of the classification layer using only few data from the target user. Similarly, Cruciani F. [30] used NN based on a multilayer perceptron (MLP) and adapted the model by updating the network weights on the user-specific data. In contrast to the CNN approach, where the input is the raw signal, Cruciani extracted features from the sensor signal and used those as the input to the network. In their work, they performed personalization by adding the user-specific data to the data of the population, and then training the model. This has the drawback of needing to have the data of the population available, which may often not be the case in real-world applications. In addition, they evaluated their methodology on datasets that were collected in a controlled manner and environment. Amrani H. [31] compared the performance of personalization through adapting CNN models and the Learn++ algorithm. They tested two neural networks: a residual neural network (ResNet) and a simplified convolutional neural network (S-CNN). For the Learn++ algorithm they used CART as the base classifier. In their work, they showed that neural networks personalize faster than the Learn++ algorithm. They demonstrated that both CNNs adapt faster to the new user than the Learn++ algorithm. The datasets they evaluated their work on were also collected in a controlled manner.

Research that exploits user similarities generally defines a metric of similarity, whether it is in the physical or demographic characteristics of the users or in the sensor signal. Then, they train the models with data from the most similar users. Ferrari A. [32] described a subject as a feature vector in which the features consist of physical properties, signal features, and a combination of both. They then compared the performance of general models (subject-independent in their text) trained on randomly chosen users and the most similar users based on the similarity between the feature vectors. Afterwards, they performed the same experiments but they added user-specific data to the training set (hybrid dataset in their text). They showed that choosing similar subjects for training leads to higher accuracy than training on dissimilar subjects. They also showed that using a hybrid dataset (i.e., adding user-specific data to the training set) also leads to better performance. For this approach, however, one has to have all the data available in the very beginning, both from the population and the user-specific data. Cruciani F. [30] adopted a similar approach for training the initial general models. They identified a subset of similar users based on similarity using a clustering procedure. They performed personalization on general models by means of updating the network weights on the user-specific data. They started with general models trained on five and ten users and succeeded in showing that personalization leads to an improvement in accuracy. They used data from accelerometer and gyroscope signals from smartphones. While they evaluated their work on a real-world dataset [33], they excluded data points that they considered to be mislabeled. They gave two examples: a flat signal with no variation that was labeled as walking or running; and they calculated cadence in walking/running labeled samples and dropped those that did not correspond with the expected cadence.

### 2.3. HAR in Real-World Scenarios

Even though there has been extensive research on HAR in many application aspects, e.g., equipment, techniques, and personalization, the majority of this research has been validated on data collected in laboratory settings or when the activities were performed in a planned manner. According to a survey from Zhang S. [34], some of the most often used benchmark datasets are OPPORTUNITY [14], UCI-HAR [35], PAMAP2 [36], WISDM [15], UTD-MHAD [37], MHEALTH [38], and USC-HAD [16].

Even though the above datasets cover many different ADL types, hand gestures, and sports activities and are performed by gender- and age-diverse participant groups, all data collections are planned and the activities are intentionally performed. This eases the labeling process, as the start and end moments of the activities are known. The activities are also clean, meaning, for example, that in the samples of walking, there are no abrupt stops. When people perform activities on command, even if instructed to do them in a natural way, the execution of these tasks might differ from the execution within their daily lives. The activities performed in this way are also uninterrupted, whereas in the real world, this is often not the case, e.g., walking on the street, during which people stop at red lights, look for things in their backpack, increase and decrease their speed according to obstacles encountered, etc. Additionally, in real-world scenarios, there is a higher chance of diverse/noisy data due to differently/incorrectly placed sensors.

The ExtraSesnsory dataset [33] is a publicly available dataset whose data were collected in real-world scenarios and whose labeling was based on self-reporting. In the paper, the authors list four conditions that have to be met for a dataset to be considered real-world (they use the terminology in-the-wild). These conditions are naturally used devices (devices that people use in their daily life and that do not restrict or change their behavior), unconstrained device placement, a natural environment, and natural behavioral content. Few methodologies have been validated on this dataset. However, this dataset has several drawbacks that make the interpretation of the results and comparison with these methodologies difficult. These drawbacks include: inconsistent labeling (e.g., labels sampled as both going upstairs and downstairs at the same time), mislabeling (e.g., flat signals while the example was labeled as walking/running [30]), wrongly sampled sessions (e.g., missing accelerometer datapoints in 20 s sessions).

In Table 1, we summarize the benchmark datasets often used in the literature. We mention the number of subjects that participated in the data collection, the devices used for the data collection, the number of activities labeled, the way the data were labeled, and the type of environment and execution of the activities. The last three aspects are very different in laboratory or controlled data collection versus RW data collection. As such, an observed execution means that the researcher was present and observed the execution of the activities; a video recording means that the execution was recorded and labeled afterwards based on these recordings; and self-reporting (which often happens in RW data collection) means that the participants were annotating by themselves the periods in which they performed certain activities. We also make the difference between controlled and uncontrolled environments, where a controlled environment would be a single room, street, desk, etc., where all of the participants perform the activities, and an uncontrolled environment would be an environment of the participant’s choosing, possibly different every time they perform an activity. Protocol and free refer to the way the activities are performed, where protocol is a predetermined sequence and/or duration of performing the activities, and free means the participant was free to choose when they performed the activity. Even more, free means the execution did not happen upon request but rather was performed normally in a real-life scenario, and only the period of execution was annotated.

We argue that research performed and evaluated on data collected in a laboratory or a controlled environment and the associated results give limited insight into the performance of these models in daily life situations, where noisy and missing sensor data are prevalent and people perform activities more naturally, interacting with their context and often combining activities.

## 3. Datasets

In this section, we describe the two datasets we used to train and evaluate our models. The first one, the IDLab dataset, is a self-collected dataset that fulfills all the requirements for RW applications: the sensor used is a wrist-worn accelerometer, which reflects the reality of what many people use in their daily lives; the data collection happens spontaneously upon the participants’ decision and in the environment they find themselves in at the moment (home, work, outdoors, …); the data labels are provided by the participants; and the whole process is neither observed nor filmed, such that the participants can act naturally. The second dataset is the MHEALTH dataset [38], which is publicly available. We consider this dataset to not meet all the requirements for RW applications as participants were asked to perform activities for a fixed amount of time or in repetitions while being filmed. We therefore consider this dataset to have been collected in a controlled manner.

### 3.1. Idlab Dataset

#### 3.1.1. Equipment and Devices

In this research, we used the Empatica E4 wearable device. E4 is a wristband equipped with an accelerometer and temperature, GSR (galvanic skin response), and PPG (photoplethysmogram) sensors. While all the sensor data were collected, we only used the accelerometer sensor for the activity recognition. We developed a smartphone application through which the E4 data were streamed to a server for storage and processing, and on which participants could register and label their activities.

#### 3.1.2. Preliminary Data Collection and Supporting Classifier

The initial data collection involved three participants, two males and one female, aged 23 to 27. They were given the E4 device, installed the smartphone application, and were given the liberty to go about their daily lives. They were given the freedom to connect the E4 and collect and label data whenever they wanted. All the added activities appeared in a timeline in the app, and they could still correct or delete the labels in case they made a mistake. As such, we have collected very diverse data for all activities: for example, a sitting activity could be working on a computer, reading a book, or watching a movie, and standing could be waiting in a queue, washing dishes, cooking, etc. In this initial data collection, we collected the following activities: cycling, running, sitting, standing, lying down, and walking.

Subsequently, we trained a gradient boosted tree model with the data we had collected. For this initial model, we adopted a traditional ML approach: using human engineered features, extracted from the three accelerometer signals (one for each axis) and their Euclidean norm (EN). The ML pipeline was as follows: We started by scaling the signals to be between −2 g and +2 g, which is also the dynamic range of the accelerometer sensor. We proceeded by applying a band-pass filter to all the signals and segmenting the data into windows of 15 s with 50% overlap. For each window, we extracted the time domain, such as the mean, minimum, maxim, kurtosis, and spectral features such as the dominant frequency. In total, we extracted 42 features. All of these features are widely used in the literature [4,5]. Finally, we standardized the features by removing the mean and scaling to unit variance. For tuning the number of trees, learning rate, depth of tree, and L2 regularisation, we performed a leave-one-out CV. The final model is trained on the full dataset. This model was deployed during the second, larger, real-world data collection. The predictions from this model then appeared in the timelines of the participants in order to lower the labeling burden. They moreover helped with recalling what the participants had done in case they decided to label it some time after they had performed the activity.

#### 3.1.3. Idlab Real-World Dataset Collection

**Figure 1:**
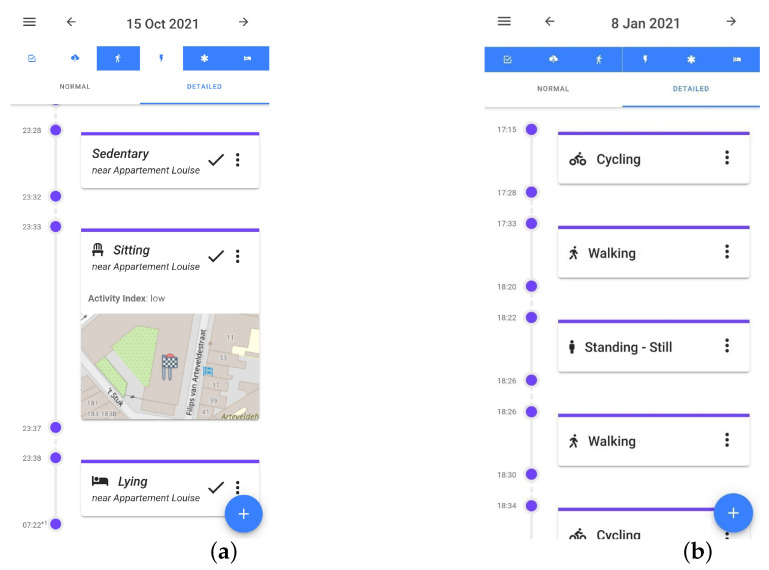
Activity timeline in app. (**a**) Predictions in the timeline. The check mark indicates they are not interacted with. When the prediction is clicked on, a map appears to show the location; (**b**) Activity labels after predictions are interacted with or manually added. There are no check marks and the activities are more specific.

**Figure 2:**
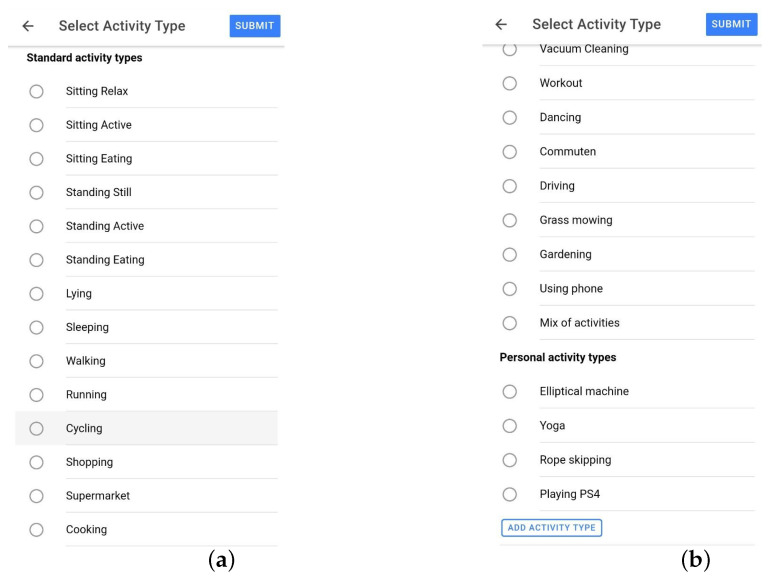
Activity list. (**a**) Part of the standard list of activities from which the user can choose. Note this is not the exhaustive list we have provided, the user may remove some labels; (**b**) Continuation of the standard activity list and personal activity list with activity labels added by the participant.

**Figure 3:**
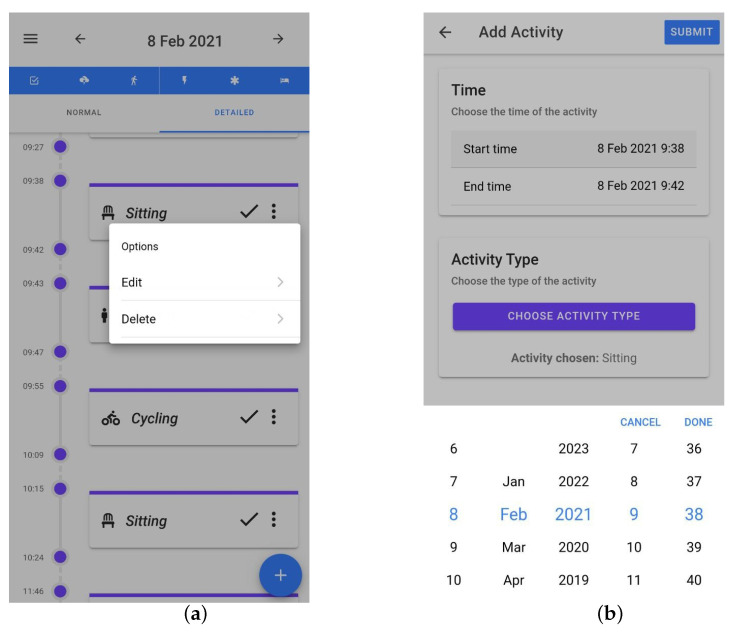
Editing a prediction or adding an activity label interface. (**a**) Clicking on the check mark confirms the prediction. Clicking on the three dots opens a menu with options to edit or delete the prediction; (**b**) Interface for editing a prediction or adding a new label.

Data were collected from eighteen participants, aged 22 to 45, consisting of five females and thirteen males. Similarly to the first data collection process, they were given the E4 device and installed the application on their own mobile device, and they had no restrictions and could go on about their daily lives. They were free to connect the E4 and collect and label data whenever they wanted. We also added several features to the application that were meant to increase the quality and decrease the loss of data: the application notified the participants when the E4 was not worn tight enough or when the Bluetooth connection was broken; data were collected continuously and were sent to the server only when the person had a wifi connection; when there was no wifi connection, we buffered all the data on the smartphone and sent them once a connection was available. To facilitate easy and, as much as possible, correct labeling, the participants received activity predictions every 5 min in the smartphone application, which were made by the model we trained and which ran on our server. The participants were free to choose whether and when they interacted with the predictions. They could confirm, correct (label and/or time), or delete the prediction. For additional recall help, we added a feature where when they clicked on a prediction, a small map appeared and showed their location at the beginning and at the end of the prediction period. Figure 1a shows how the predictions appeared in the timeline. The check mark indicates that it is a prediction that has not been interacted with and can be used to confirm a prediction. When labeling, participants could choose an activity from the standard activity type list, which was a list of activities added by the researchers. However, participants could add their own labels to a personal activity types list. These lists are shown in Figure 2a,b. Figure 3a shows the options of editing and deleting predictions, and Figure 3b shows the interface for choosing a start/end time for an activity label.

As such, our dataset fulfills all the conditions for a dataset to be considered real-world, as defined by Vaizman Y. [33]. In comparison to the ExtraSensory dataset, our dataset contains continuously sampled sensor data (versus only 20 s each minute), and our smartwatch sensor data are sampled correctly (no missing samples when connected to the device) and in a time-ordered manner.

#### 3.1.4. Activities

For the data collection campaign, we asked participants to label different activities, all very common in people’s daily lives: *sitting* (while using a computer), *standing still*, *walking*, *running*, and *cycling*. They could also label other activities, such as cooking, doing groceries in a supermarket, and commuting, or add activities of their own interest, as shown in Figure 2a,b. In this research, we focused on the first five mentioned for several reasons: we had a decent amount of data from these activities; they are some of the most commonly used and evaluated in the literature; and we were positive that they are detectable with a single wrist-worn accelerometer.

#### 3.1.5. Correcting Overlapping Labels

**Figure 4:**
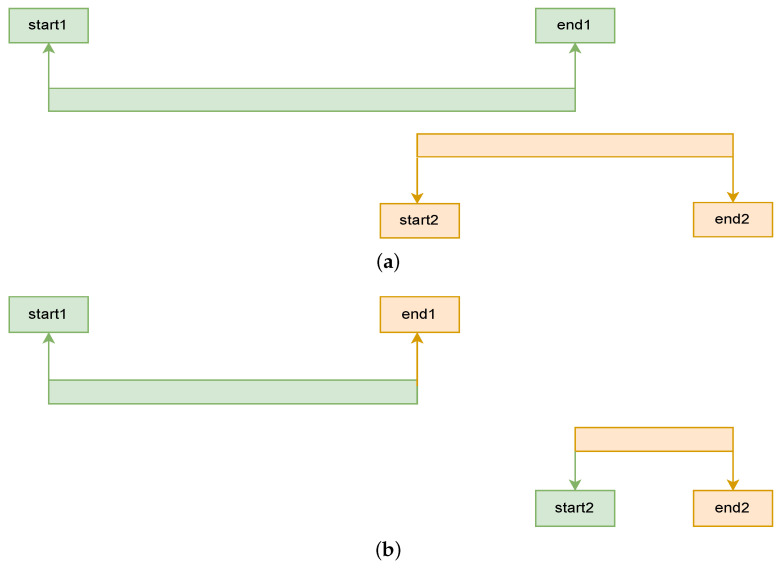
Solution to overlapping labels. (**a**) Initial situation; (**b**) Situation after readjusting the start and end times.

Since the data collection was neither guided nor observed, there were mistakes in the labels that we first had to correct, namely, overlapping labels: a period of time that is labeled with two different activities, as illustrated in Figure 4a. It was important to address and correct these since they would otherwise lead to samples appearing several times with different labels, deteriorating the learning process. This mistake was often made as a result of choosing the incorrect option from the list of activity labels or wrongly indicating (or correcting) the time. As such, we cannot speak of multi-labeling, i.e., executing multiple activities at the same time, because the activities were mostly impossible to execute simultaneously (e.g., walking and sitting). To correct this, we first ordered all the activities based on start times. Once we found overlapping labels, we adjusted their start and end times to exclude the overlapping time range from both, as shown in Figure 4b. In the cases where the duration of an activity was fully within the duration of another one, we restricted the end time of the longer activity to the start of the shorter activity. The shorter activity was afterwards fully removed. This led to the loss of (possibly valuable) data.

#### 3.1.6. Final Dataset

The total duration of labeled data (as given by the participants and before removing overlaps), grouped into each of the five activities we focused the data collection on, is given in Table 2, together with the amount of usable labeled sensor data after cleaning and the number of labels given in the app by the participants. The first two are not the same due to disconnected wearable device intervals and thus missing data and/or mislabeling.

### 3.2. MHEALTH Dataset

The MHEALTH dataset [38] consists of data collected by 10 subjects in an (according to the researcher who carried out the data collection) out-of-lab environment. The subjects wore wearable sensors on several parts of their bodies, including the right wrist. The data were sampled at a rate of 50 Hz, but we downsampled the signal to 32 Hz (using the Pandas resample method with mean aggregator) so that it corresponded to the sampling rate of our dataset. Each subject performed 12 different activities, either for one minute each or with 20 repetitions per activity, depending on the nature of the activity. Each session was recorded with a video camera. The subjects were not given any constraints for executing the activities, except that they had to try their best. In this research, we selected four activities from the MHEALTH dataset, namely cycling, running, standing still, and walking, as they intersect with the activities we collected. In the selected subset, we had in total ten minutes per activity (one minute of activity per person).

## 4. Methodology

In this section, we first describe the methodology for building the general models for the IDLab dataset, followed by the general models for the MHEALTH dataset. In the last subsection, we explain the personalization methodology, which is the same for both datasets.

### 4.1. General Models—Real-World IDLab Dataset

In this section, we explain the approach for building the general models. As mentioned in the previous section, we considered five low-level activities, namely: sitting (using a computer on a table), standing still, walking, running, and cycling. We built several general models for each participant. A general model for participant X was trained on data from all the other participants (namely, participants 0, 1, …, X − 1, X + 1, …, and 17). Each model was trained on a different amount of data. We did this to research the amount of data at which we reached the maximum accuracy (a point after which it makes little sense to continue collecting data). We looked at both situations: improving the general models, but also improving the personalized models. We added the data in a time-ordered manner. This follows the nature of collecting data in RW setting. In the following subsections we give details of this approach.

#### 4.1.1. Processing the Sensor Data

We first scaled the signals so that they had values between −2 and +2, which represent acceleration between −2 g and +2 g. We then segmented the data into 12 s windows with 50% stride. Given the 32 Hz accelerometer sampling rate, a single training sample was thus a 3-by-384 matrix (3 is from the X, Y, and Z axes from the accelerometer, and 384 is from 12 s of 32 values per second).

#### 4.1.2. Personalization and Hold-Out Test Set

**Figure 5:**
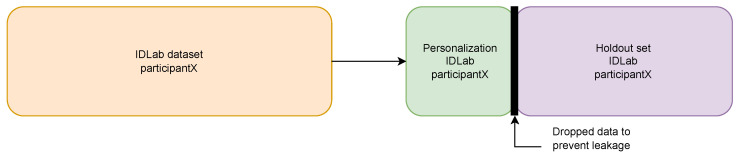
Split of data in personalization and hold-out sets. This approach is used on data from each activity for each participant in the dataset.

Each participant’s data were partitioned into a personalization set and a hold-out test set. The personalization data were chosen to be small and fixed for each activity across participants. We chose the amount of personalization data based on what we found to be reasonable to require from a user in a RW scenario and what we considered to be enough to capture personal differences in each activity. As such, we chose the following amounts for the activities that we considered in this research: computer table—120 min, cycling—10 min, running—10 min, standing still—5 min, and walking—10 min. We also required at least half of this amount of additional data for testing. In case a participant had labeled less than this minimum amount, we did not consider that activity for that person. Additionally, to avoid information leakage, we dropped 2 min of data at the splitting point for each activity. The hold-out test set was used to evaluate every model that was trained for that participant (general IDLab model, personalized IDLab model, general MHEALTH model, and personalized MHEALTH model). Figure 5 is a visual representation of how the data was split into the personalization and hold-out sets. This was done for each activity within the data for each participant. Table 3 shows the amount of samples in the personalization set and the hold-out set for each participant.

#### 4.1.3. Splitting Data in Time-Ordered Chunks

**Figure 6:**
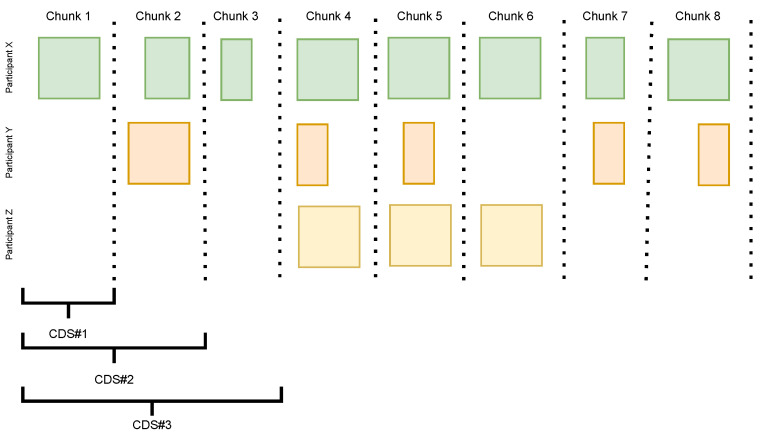
Visualization of the chunks and the cumulative data sets (CDS). Possible data situation from only three participants. Note how the first three CDS do not contain data from participant Z.

Each general model for each participant was trained on the full dataset from all other participants. To test the impact of using more data for training, we split the data from all the participants in 8 time-ordered chunks. The chunks follow each other absolutely in time, meaning the data in chunk 1 precede the data in chunk 2, which in turn precede the data in chunk 3, and the time split is the same for all participants. This leads to empty chunks for certain participants (e.g., the early chunks, if the participant started collecting data later). The chunks are not necessarily continuous, as there may be gaps in the data. The 8 chunks of data were used to construct 8 *cumulative datasets* (CDSs). Each CDS is incrementally larger than the previous one. CDS #1 is made of chunk 1. CDS #4 consists of chunks 1, 2, 3, and 4. Figure 6 visualizes the chunking and the construction of the CDS.

Table 4 shows the number of samples in each CDS per activity across all participants.

It can be noted that there is no equal increment in data for each subsequent CDS. We consciously chose to follow an approach that is realizable in a real-world setting, and have thus performed the chunking according to an absolute time split across all participants.

#### 4.1.4. General Models

We trained eight general models for each participant, one per CDS, using the data from the other participants (leave-one-person-out evaluation approach). These general models were used later as a starting point for personalization by means of transfer learning, as explained in Section 4.3. There were several participants who do not have data in chunks 1, 2, and 3 (some even until later). Naturally, for such participants, we would use the full CDS to train their general model, i.e., if a participant has no data of their own in a CDS, then the CDS without their own data is just the whole CDS. So, if multiple participants have no data in the CDS, their general model would be trained on the same full CDS. This is the situation in our hypothetical situation illustrated in Figure 6. Participants Y and Z do not have data in CDS#1. Instead of retraining each model several times with the full CDS, this model is trained only once and reused across such participants. In our example, that would be one general model for participants Y and Z, trained on data from participant X. This model is then evaluated on each of those participants’ data separately.

#### 4.1.5. Architecture

**Figure A1:**
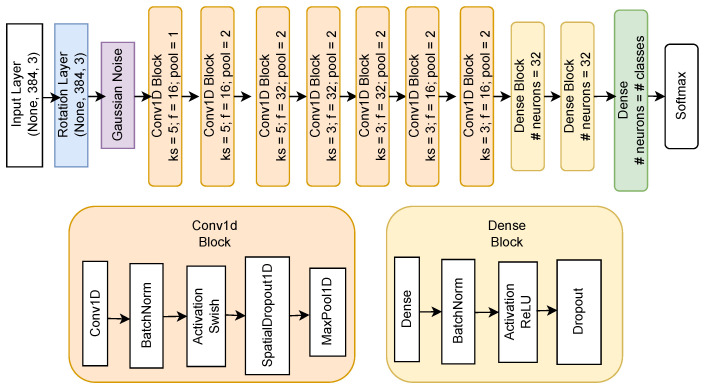
Visualization of the CNN model architecture. Abbreviations: ks = kernel size; f = filters; pool = pooling.

For the general models, we chose a CNN model with 7 1D convolutional layers, 2 dense layers (32 and 16 neurons), and a softmax layer. The CNN architecture was selected due to its parameter efficiency and being an intuitive choice for identifying temporal patterns. The number of convolutional layers and their kernel sizes provide a perceptive field of 3.5 s, which is a time length we considered sufficient to detect the different types of activities. We also added a *rotational layer* right after the input for data augmentation. This layer performs small rotations by constructing a random 3×3 rotation matrix (i.e., determinant 1) by performing Gram–Schmidt orthogonalization on a matrix of which the elements are randomly sampled from a normal distribution. The strength of the rotation can be dialed back by making a barycentric combination between the 3×3 identity matrix and the randomly sampled matrix before starting the Gram–Schmidt algorithm. This augmentation mimics having the wristband placed slightly differently on the wrist and thus makes the model more robust to participants wearing it slightly different across sessions. In the convolutional layers, we used the swish activation function, and in the dense layers, the ReLU. The ReLU and swish activations were selected due to their proven efficiency in training deep models [39]. For regularization, we used a spatial dropout between the convolutional layers and a normal dropout between the dense layers. We also added Gaussian noise to the input data and to the weights of the first two convolutional layers. These are all known methods for preventing overfitting [40,41]. Figure A1 in Appendix A visualizes the model architecture. Due to time and computational limitations, we determined the hyper-parameters by performing a limited search using a small subset of data from two participants. We were satisfied once we found a stable combination that led to convergence. These parameters are by no means optimal, neither for the general nor for the personalized models. We kept all the hyper-parameters: 105 epochs, learning rate of 0.0008 with the Adam optimizer, spatial and normal dropout rates of 0.15, Gaussian noise of 0.03, rotation strength of 0.2, and batch size of 600. We also set a scheduler for lowering the learning rate every 30 epochs by a factor of 0.3, which helps to reach convergence.

### 4.2. General Models—Controlled MHEALTH Dataset

**Figure 7:**
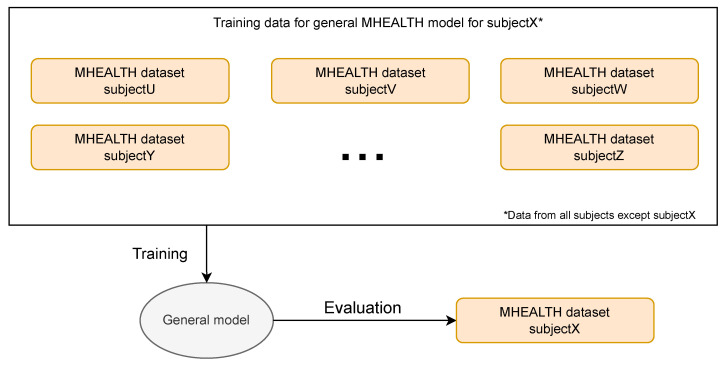
Flowchart of training and evaluating general models with MHEALTH data.

Similarly, we trained ten general models, one for each subject, on the MHEALTH dataset. We did not personalize MHEALTH data, so we did not split them into personalization and hold-out sets per participant. We only performed a leave-one-subject-out evaluation on the full data of the evaluating subject. Since there is only one minute per activity per subject in this dataset, we did not perform chunking either. We kept the architecture of 7 1D convolutional layers, 2 dense layers, and a softmax layer. Due to the limited amount and the nature of the data, we changed some of the hyper-parameters. We obtained the hyper-parameters by performing an inner leave-one-out cross validation for each general model. We realized that the hyper-parameters for each model were relatively close to each other, so, for simplicity, we decided to make them fixed. They are as follows: 270 epochs, learning rate of 0.005, spatial and normal dropout rates of 0.075, Gaussian noise of 0.015, rotation strength of 0.1, and batch size of 600. We also set a scheduler for lowering the learning rate every 50 epochs by a factor of 0.3. We finally trained a model with data from all 10 participants. This model was later evaluated on the real-world data. Figure 7 shows the flowchart of training and evaluating the general models with and on MHEALTH data.

### 4.3. Personalization

**Figure 8:**
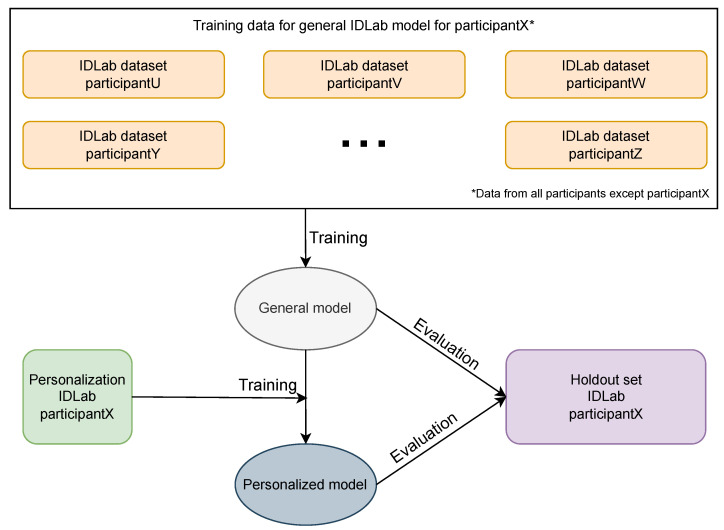
Flowchart of training and evaluation of general and personalized models with RW data.

**Figure 9:**
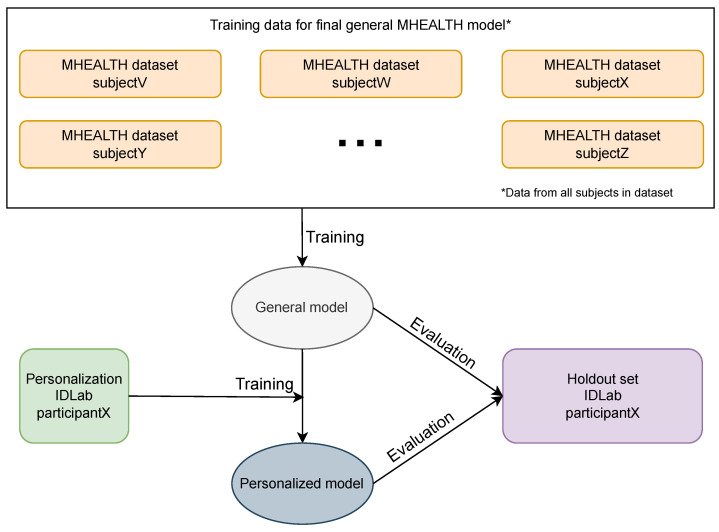
Flowchart of the training of the general MHEALTH model and the evaluation of it on RW data, and additional personalization of it and evaluation of it again with RW data.

We performed personalization using transfer learning by freezing the first 3 convolutional layers and continuing to train the last 4 convolutional layers. The dense layers were kept frozen. The same personalization strategy was used for the general models trained on both the RW IDLab dataset and the controlled MHEALTH dataset. Whereas we personalized models from both the RW IDLab dataset and MHEALTH dataset, we used only personalization data from the RW IDLab dataset. To test the effect of personalization, for each participant, we personalized each general model that was trained for the given participant. We moreover personalized each model five times by training on different portions of the personalization data: 20%, 40%, 60%, 80%, and 100%. We did this to analyze the impact of the amount of data on the accuracy of the personalized models. We additionally made small changes in the hyper-parameters, keeping in mind that we were training on few data: we used an SGD optimizer and accordingly increased the learning rate; we increased regularization by increasing the Gaussian noise and the dropout rate. Figure 8 shows the training of general and personalized models using RW data and the evaluation of them on RW data. Figure 9 shows the flowchart of training the final general MHEALTH model and the personalization and evaluation using RW data.

## 5. Experiments and Results

In this paper, we performed several experiments to answer the following questions:

1. Can we use a single wrist-worn accelerometer for HAR in the real world?
2. How does the predictive performance of the general models improve with the increase in the amount of training data?
3. How much personal data do we need for the improvement of a general model (not trained on the same person)?
4. How much data do we need for the general model to generalize so well that it makes little sense to personalize?
5. How well do models trained on data collected in controlled environments perform in RW scenarios?
6. Can the personalization of models trained on data collected in controlled environments with RW data lead to satisfying performance?

In each subsection, we show the results of each experiment that helped us to answer these questions.

### 5.1. Evaluation Metrics

In our research, we used the following evaluation metrics: *F1-micro*, *F1-macro*, *F1-weighted*, *balanced accuracy*, and *logloss* (or cross-entropy loss). Before we give the definitions of these metrics, we give the definitions of *precision* and *recall*.

$$Precision=\frac{TP}{TP+FP}$$

$$Recall=\frac{TP}{TP+FN}$$

*F1-micro* is a measure of the overall accuracy, where the precision and recall of each class are calculated globally (across all classes) rather than independently. *F1-macro* measures the average accuracy, where the precision and recall of each class are calculated independently and then averaged together. *Balanced accuracy* is defined as the average of the recall for each class

$$F1_{micro}=2*\frac{\left(Precision*Recall\right)}{\left(Precision+Recall\right)}$$

$$F1_{macro}=\frac{1}{C}\sum_{i=1}^{C}2*\frac{\left(Precision_{i}*Recall_{i}\right)}{\left(Precision_{i}+Recall_{i}\right)}$$

$$BalancedAccuracy=\frac{1}{C}\sum_{i=1}^{C}Recall_{i}$$

$$logloss=-\frac{1}{N}\sum_{i=1}^{N}\sum_{j=1}^{C}y_{i,j}log\left(p_{i,j}\right)$$

where *C* is the number of classes, *N* is the number of samples, and $y_{i,j}$ is the true label of sample *i* for class *j*. $p_{i,j}$ is the predicted probability of sample *i* for class *j*.

### 5.2. General Models—RW IDLab Dataset

In this section, we present the results obtained with the general models trained on RW data. Each of these models was trained on the data from the other participants who had data available in the given CDS. The models were evaluated on the hold-out set of the evaluating participant. In Table 5 we show the results averaged across all participants per CDS.

**Figure 10:**
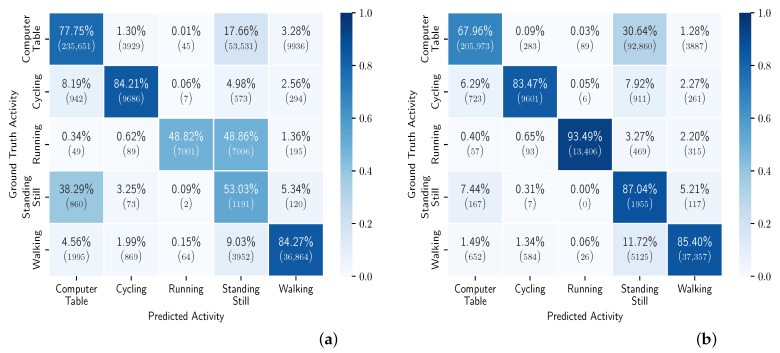
Confusion matrix of general models, across all users, trained with data from CDS #1 and CDS #8. (**a**) CDS #1; (**b**) CDS #8.

**Figure 11:**
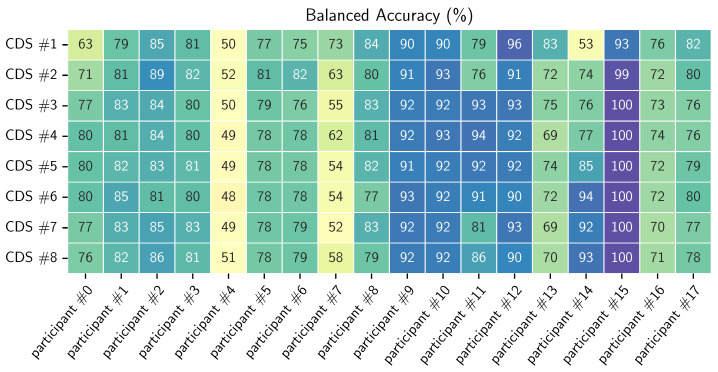
Balanced accuracy for each participant for each CDS.

The results of the general models for each participant can be found in Appendix B. Figure 10 shows the confusion matrices of the models trained on CDS #1 and #8. We can see that there is notable improvement in the running and standing still classes. This may be due to the very limited amount of training samples in these two classes. From the second chunk on, the improvement is minimal, when looking across all participants. There are, however, differences when looking at the individual results. For example, in Figure 11, we can see that we do not always observe a monotonic increase in balanced accuracy with the increase in CDS.

### 5.3. Personalization of the General RW Models

**Figure 12:**
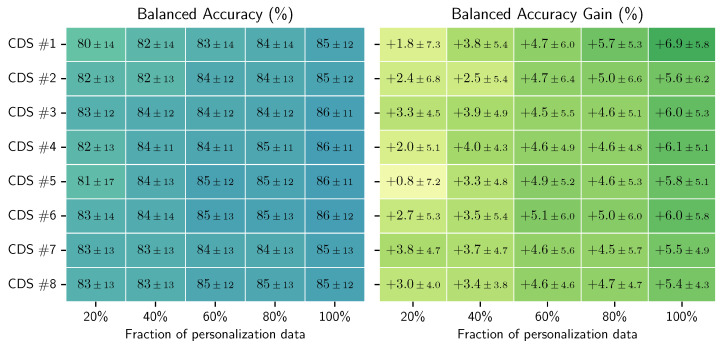
Balanced accuracy and gain in balanced accuracy of the personalized models per CDS and percentage of available personalization data used. Reported values: mean and standard deviation.

**Figure 13:**
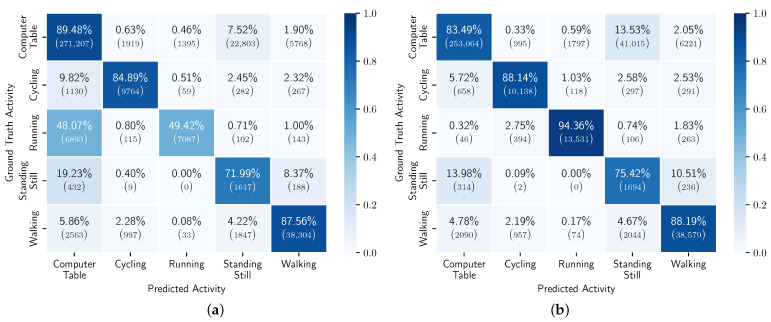
Confusion matrix of personalized models, across all users, transferred from models with data from CDS #1 and CDS #8 and trained with 100% of the personalization data. (**a**) CDS #1; (**b**) CDS #8.

**Figure 14:**
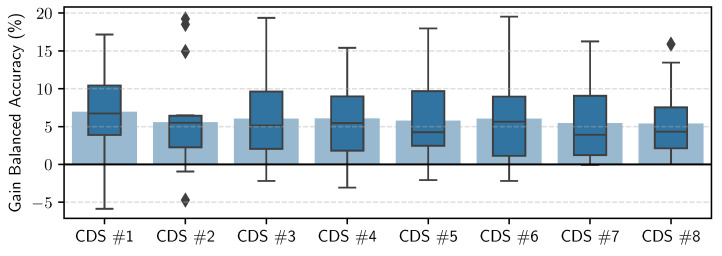
Boxplots of the gain in balanced accuracy when personalizing with 100% of the personalization data, per CDS, averaged across all participants, with the blue shadow indicating the mean.

**Figure 15:**
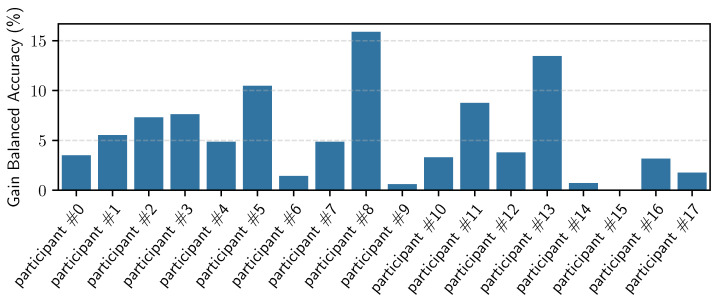
Bar plot per participant of the gain in balanced accuracy when personalizing the model trained on CDS #8 with 100% of the personalization data.

In these experiments, we personalized each general model (for each of the eight CDS) five times, namely, with 20% to 100% of the personalization data (personalization set). Each model was evaluated on the hold-out set for the given participant. Figure 12 shows the balanced accuracy results per CDS, averaged across all the participants. We can see that we obtain up to 6% improvement when personalizing. We can also see that the improvement gain is less the more data we have for training the general models, i.e., the improvement is less for CDS #8 compared to CDS #1. Figure 13 shows the confusion matrices for the personalized models across all users, using 100% of the personalization data, for CDS #1 and CDS #8. Figure 14 shows the boxplots and the mean (blue shadow bar) of the gain in balanced accuracy between general and personalized models with 100% of the personalization data. We can observe that, for the majority of the participants, the personalization has a positive impact and improves the balanced accuracy. There are few participants for who the personalization has a negative impact. Figure 15 zooms in on the gain in balanced accuracy for each participant when personalizing the general model trained on CDS #8 (the most general data) with 100% of the personalization data. Even though for few of the participants the gain was marginal, we observed a positive impact for all of the participants, three of them more than 10%.

### 5.4. General Models—Controlled MHEALTH Dataset

**Figure 16:**
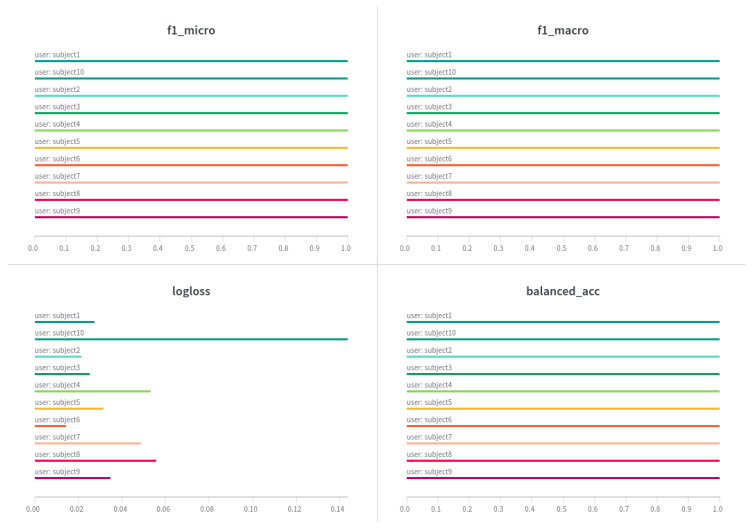
Results of the general models for the MHEALTH data.

We also trained models using the publicly available MHEALTH dataset. In these experiments, we trained one general model for each participant, following a leave-one-user approach. This meant that we trained ten different models, each trained on data from nine participants and evaluated on the hold-out participant. For each hold-out participant, we obtained a 100% balanced accuracy. Figure 16 shows the results we obtained with these models.

### 5.5. RW Data Versus Controlled Environment Data

The results of the cross evaluation are shown in Table 6 and Table 7. The first shows the general models trained on either the RW data or the MHEALTH data, and evaluated on the MHEALTH data. The second shows the same general models with both evaluated on the RW data. We can see in Table 6 that even when using only the first five CDS, we achieve almost perfect results. The results of evaluating the models on RW data are quite different. Whereas the models trained on RW data perform well (as already seen in Section 5.2), the model trained on the MHEALTH data performs poorly and achieves only 62% balanced accuracy.

### 5.6. Personalizing MHEALTH Models with RW Data

**Figure 17:**
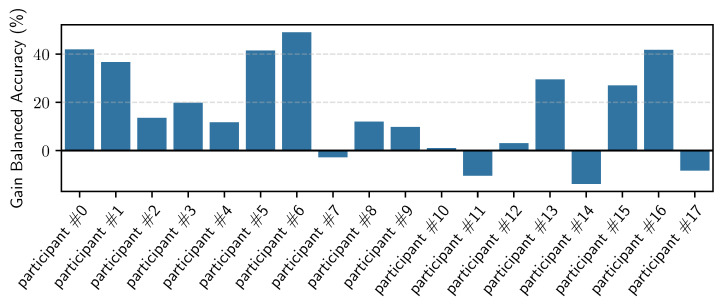
Bar plot per participant of the gain in balanced accuracy when personalizing the general MHEALTH model using 100% of the personalization data.

Similarly to personalizing the different general models trained with RW data, we personalized the MHEALTH model using the personalization data of each participant of the RW dataset. The results are shown in Table 8. We observed that we achieved a mean improvement of 14% of balanced accuracy when using 100% of the available personalizing data. Even if there is no significant difference in the F1 and accuracy metrics when using fewer personalizing data, we still see a decrease in the logloss, which indicates that the model becomes more certain in distinguishing the activities. Figure 17 shows the gain in balanced accuracy after personalization per participant. We can observe that, for the majority of participants, there is improvement. However, there are four participants for whom we observed a decrease in the balanced accuracy. While we do not have a certain explanation of why this is the case, we have several possible explanations: the general model of MHEALTH is very fragile on its own, and based on the large standard deviation reported in Table 7, we can conclude that it performs very well for certain participants and very poorly for others. Personalizing such a model with few data may then move the decision boundaries in unexpected and suboptimal ways. Another possibility is that part of the personalization data is mislabeled or not representative of the data for that user. Whereas this may not pose a significant problem when personalizing a robust general model, like the general models trained on RW data, it might be an issue when personalizing a fragile model such as the general model trained on the MHEALTH data.

## 6. Discussion

The main goal of this research was to show the importance of training and, most importantly, evaluating machine learning models on real-world data. Current research develops and evaluates techniques and methodologies mainly on laboratory-collected data or data collected in a controlled environment. The models trained on laboratory-collected data rarely achieve the same results in real-world scenarios. To achieve satisfying performance in real-world scenarios, research that is performed on real-world collected data is needed. There is need for methodologies and techniques that can cope with the challenges that the real world brings, such as interleaved activities, mislabeling, interruptions in activities, imbalanced and missing data, etc. In our work, we mainly focused on answering six questions which were stated in Figure 5. In this section, we briefly discuss our findings that helped us to answer these questions.

**Can we use a single wrist-worn accelerometer for HAR in the real world?**

In this research, we showed that it is possible to perform HAR on basic activities in the real world by using a single wrist-worn accelerometer and with data collected in a fully uncontrolled environment. We presented a coping technique, which is explained in detail in Section 3.1.5, for doubled and incorrect labeling, meaning two different labels for the same period of activities that are impossible to be performed at the same time. This technique leads to discarding data, which leaves open space for researching different coping techniques for this situation. For all experiments we use convolutional neural networks as machine learning algorithm. Due to time and computing limitations we keep the architecture and hyper-parameters fixed for all (sub)experiments. This may lead to suboptimal results, but the results we obtained were sufficient in achieving the goal of this research. We achieved a mean of 80% of balanced accuracy for the general models across all participants. The full results can be found in Table 5 and Figure 10 and Figure 11.

**How does the predictive performance of the general models improve with the increase in the amount of training data?**

Even though, on average, we could see slight improvement in the results (presented in Table 5), there was a rather big difference across participants. This can partially be the result of not fine-tuning the hyper-parameters for each model, but it can also be the result of introducing mislabeled data in the particular CDS on which the model was trained. Another possible reason is the introduction of data that are different from the data of the participant on who we evaluated (inter-participant differences in executing the activities). When evaluating this approach on the data collected in controlled environment, we observed clear improvement in the first four data increments: we see in Figure 6 results starting from 70% in CDS#1 and reaching 100% in CDS#8 of balanced accuracy, which was maintained in the rest of the evaluations.

**How much personal data do we need for the improvement of a general model (not trained on this person)?**

We showed that personalization improves the predictive performance of the RW models with few personalization data. The highest average gain across users we achieves was 6%. The full results can be found in Figure 12. Whereas the personalization of models based on accelerometer data may lead to a great improvement when the target user performs the activities in a different way to the population on which the general model was trained (e.g., elderly people walking with help of a wheeled walker vs. an active adult walking), the gain in performance seemed to be marginal when the target user and the population have similar physical and demographic properties. This marginal difference for certain participants, as seen in our results, may be the result of the model learning the user’s labeling bias. This means that some users will ignore short interruptions of their activities (waiting at a red light while walking or cycling or going to grab a glass of water during work/using a computer session) and will include these periods in their labels, whereas other users will exclude these periods from the labels or will label them with the “true” activity. Another challenge when personalizing on RW data is unintentional mislabeling. In our research, we did not apply sample selection for the data that we used for the personalization; however, among these samples, there might be mislabeled data, which may have a negative impact on the learning process. This may explain why in Figure 14 we see that the whiskers go below zero when personalizing the models trained on fewer data (lower CDS). This is not the case when we personalize models trained on more data (higher CDS).

Future research can focus on data selection for personalization.

**How much data do we need for the general model to generalize so well so that it makes little sense to personalize?**

We can see in Figure 12, that as we increase the data used for training the general models, the less improvement we see after personalization. However, we obtained the best results when personalizing the general models that were trained on fewer data.

**How well do models trained on data collected in controlled environments perform in RW scenarios?**

We demonstrated that, while one can obtain perfect results on data collected in a controlled manner, these models do not work well in real-world scenarios. Our evaluation, as presented in Table 7, showed a decrease in balanced accuracy of 22% on average. We argue, then, that one should be careful when developing methodologies and training models on laboratory-collected data or data collected in controlled environments that are meant to be applied in the real world.

**Can the personalization of models trained on data collected in controlled environments with RW data lead to a satisfying performance?**

We show in Table 8, that when there is no opportunity to collect and train models on RW data, personalizing a model trained on data collected in a controlled environment with little personal data collected in the real world improves the balanced accuracy by up to 17% percent on average. This means that there is a possibility to adapt and improve models trained on data collected in controlled settings for real-world applications by using transfer learning and limited data collected in the real world.

**Can personalization be used to learn new activities, that is, activities that were not present in the training of the general model but are present in the user-specific dataset?**

The personalization methodology can be adapted to be able to learn new activities that were not present in the training of the general model. We would have to use a new Softmax layer with a number of neurons corresponding to the new number of activities we are going to detect. Additional changes might be needed, such as unfreezing and training more layers, or adding additional convolutional and/or dense layers. We consider this research topic to be out of scope for this paper but possible for future work.

## 7. Conclusions

In this work, we argued that models and methodologies for human activity recognition developed on data collected in a laboratory or controlled settings have little relevance and application in real-world scenarios. We also identified a lack of publicly available RW data for human activity recognition. To that end, we collected a dataset that fulfills all the requirements for real-world applications. This dataset consisted of data from 18 participants and a variety of activities, all of them very common in daily life. We presented the challenges of working with and processing data of this character, such as mechanisms for aiding the labeling process during data collection. We additionally present a coping mechanism for mislabeling during the pre-processing of the data. We then trained general and personalized models and evaluated these with this real-word dataset and we showed that personalization has a positive impact on the predictive performance. We achieved high accuracy results, averaging an 80% balanced accuracy for the general models, and we observed an improvement of up to 6% on average. To prove the importance of evaluating on real-world data, we also trained and evaluated models with data collected in controlled settings. We then cross-evaluated these models and showed that, whereas the models trained on real-world data achieve perfect results when evaluated on data collected in controlled setting, the opposite does not hold. Namely, we observe a significant drop (22%) in predictive capability when we evaluated the models trained on data collected in controlled environments on our real-world dataset. We finally showed that, even when using little personalization, real-world data can significantly improve, up to 17%, the performance of the models trained on data collected in a controlled environment. This may be taken in consideration when an activity recognition model is needed for a real-world application, but there are no means of collecting (enough) real-world data to build general models. In such a scenario, one may opt for training a general model with a publicly available dataset that was collected in a controlled manner, and then performing a limited data collection for personalization. During this research, we detected several future work possibilities: coping (and/or automated) techniques for double or mislabeled data that avoid discarding data; an automated choice of personalization samples that will have the biggest positive impact on the performance of the personalized model; and performing similar research to this to investigate the potential and possibilities of real-world collected data for more complex and diverse activities, such as cooking, tidying up, and home workout sessions.

## Figures and Tables

**Table 1:** Benchmark datasets used in the literature and their characteristics and our newly collected dataset. Abbreviations: OE (observed execution), VR/VI (video recording/visual inspection), SR (self report) C (controlled), UC (uncontrolled), P (protocol), F (free).

Dataset	#S	Devices	#Activities	Labeling Procedure	Env. and Exec.
OPPORTUNITY	12	body, object, and ambient sensors	13 actions, 17 gestures, 5 activities	VR/VI	C and P
UCI-HAR	30	smartphones	8 activities	OE	C and P
PAMAP2	9	body sensors	12 activities	OE	C and P
WISDM	51	smartphones, smartwatches	18 activities	OE	C and P
UTD-MHAD	8	camera, body sensors	27 actions	VR/VI	C and P
USC-HAD	14	body sensor	12 activities	OE	C and P
MHEALTH	10	body sensors	12 activities	OE	C and P
ExtraSensory	60	smartphones, smartwatches	103 activities (51 after cleaning)	SR	UC and F
IDLab	18	smartphones, smartwatches	26 activities	SR	UC and F

**Table 2:** Label duration (as given by participants before removing overlaps), usable labeled sensor data after cleaning duration, and number of labels given by the participants.

Activity	Labeled Duration	Sensor Duration	Number of Labels
Computer Table	23 days 8 h 56 m 38 s	18 days 15 h 22 m 10 s	579
Walking	3 days 7 h 42 m 30 s	2 days 15 h 05 m 00 s	328
Running	1 day 4 h 53 m 42 s	21 h 17 m 15 s	122
Cycling	1 day 1 h 20 m 03 s	18 h 08 m 30 s	59
Standing Still	6 h 23 m 41 s	4 h 25 m 35 s	55

**Table 3:** Amount of samples in personalization and hold-out sets per participant (one sample is a 3-by-384 matrix, 3 is from the X, Y, and Z axes from the accelerometer, and 384 is from 12 s of 32 values per second).

	Personalization Set					Hold-Out Set				
Participant	Computer Table	Cycling	Running	Standing Still	Walking	Computer Table	Cycling	Running	Standing Still	Walking
0	1195	0	0	49	99	28,689	0	0	113	965
1	1197	97	99	47	0	39,462	2367	1021	100	0
2	1194	0	0	0	99	7716	0	0	0	2880
3	1186	97	0	0	99	33,412	1446	0	0	8533
4	1197	99	99	0	95	9954	1541	118	0	516
5	1194	99	0	0	96	45,472	274	0	0	2094
6	1194	0	0	47	99	32,827	0	0	1115	3382
7	1190	99	0	47	98	22,447	1106	0	55	4383
8	1196	99	0	0	0	6386	468	0	0	0
9	0	97	99	0	99	0	171	4036	0	2427
10	0	94	99	0	99	0	344	643	0	166
11	0	98	0	48	98	0	288	0	140	3539
12	0	0	99	0	99	0	0	273	0	264
13	1195	99	99	0	98	5062	172	1176	0	1775
14	1199	97	99	49	98	15,206	2998	6998	569	10,140
15	0	0	99	0	95	0	0	75	0	61
16	1194	0	0	49	99	41,695	0	0	154	1638
17	1195	96	0	0	99	14,764	327	0	0	981

**Table 4:** Number of samples in each CDS per activity (combined for all participants).

CDS	Computer Table	Cycling	Running	Standing Still	Walking
#1	18,508	1290	2469	546	5253
#2	31,349	2079	3083	738	13,789
#3	34,010	2830	4301	1420	21,607
#4	54,447	3898	7509	1786	24,899
#5	127,397	4733	10,169	1951	31,159
#6	195,769	6227	11,077	2111	34,586
#7	275,343	11,525	13,677	2250	40,461
#8	318,618	12,673	15,132	2582	45,313

**Table 5:** Results of the general models per data chunk, averaged across all participants.

CDS	Balanced Accuracy	F1-Macro	F1-Micro	Logloss
#1	0.78±0.12	0.47±0.12	0.82±0.10	0.62±0.37
#2	0.79±0.11	0.49±0.12	0.83±0.11	0.46±0.20
#3	0.80±0.12	0.50±0.17	0.78±0.15	0.49±0.24
#4	0.80±0.12	0.51±0.16	0.78±0.13	0.50±0.23
#5	0.80±0.12	0.51±0.16	0.77±0.16	0.49±0.24
#6	0.80±0.13	0.53±0.17	0.78±0.16	0.49±0.25
#7	0.80±0.13	0.54±0.16	0.78±0.17	0.46±0.27
#8	0.80±0.12	0.52±0.16	0.78±0.15	0.46±0.23

**Table 6:** Results of the models trained on the RW data and the MHEALTH data evaluated on MHEALTH data (four activities).

Training Data	Balanced Accuracy	F1-Macro	F1-Micro	Logloss
CDS #1	0.70±0.08	0.57±0.05	0.76±0.06	0.38±0.12
CDS #2	0.74±0.03	0.59±0.02	0.79±0.03	0.32±0.07
CDS #3	0.97±0.07	0.94±0.13	0.97±0.06	0.14±0.05
CDS #4	0.97±0.07	0.94±0.13	0.98±0.06	0.13±0.04
CDS #5	1.00±0.01	0.98±0.06	1.00±0.01	0.09±0.02
CDS #6	0.97±0.08	0.93±0.13	0.97±0.06	0.16±0.05
CDS #7	0.99±0.03	0.97±0.08	0.99±0.03	0.11±0.04
CDS #8	1.00±0.00	1.00±0.00	1.00±0.00	0.12±0.02
MHEALTH	1.00±0.00	1.00±0.00	1.00±0.00	0.05±0.04

**Table 7:** Results of the models trained on the RW data and the MHEALTH data evaluated on RW data (four activities).

Training Data	Balanced Accuracy	F1-Macro	F1-Micro	Logloss
CDS #1	0.84±0.12	0.51±0.13	0.85±0.10	0.57±0.57
CDS #2	0.84±0.12	0.51±0.14	0.86±0.10	0.46±0.36
CDS #3	0.85±0.13	0.55±0.19	0.86±0.11	0.44±0.36
CDS #4	0.85±0.13	0.55±0.19	0.86±0.10	0.46±0.39
CDS #5	0.85±0.12	0.55±0.19	0.87±0.09	0.43±0.37
CDS #6	0.85±0.13	0.58±0.19	0.86±0.10	0.41±0.30
CDS #7	0.84±0.13	0.58±0.19	0.85±0.12	0.44±0.35
CDS #8	0.84±0.13	0.55±0.19	0.85±0.11	0.45±0.30
MHEALTH	0.62±0.25	0.35±0.20	0.59±0.30	1.66±1.44

**Table 8:** Results of the general MHEALTH models personalized with RW data. Results indicate mean and standard deviation across participants.

Fraction Personalization	Balanced Accuracy	F1-Macro	F1-Micro	Logloss
0%	0.62±0.25	0.35±0.20	0.59±0.30	1.66±1.44
20%	0.70±0.20	0.54±0.23	0.66±0.24	0.88±0.51
40%	0.73±0.21	0.57±0.21	0.68±0.23	0.88±0.52
60%	0.76±0.18	0.64±0.22	0.74±0.22	0.74±0.54
80%	0.78±0.19	0.64±0.23	0.75±0.23	0.66±0.51
100%	0.79±0.18	0.66±0.21	0.76±0.20	0.62±0.46

## Data Availability

The dataset presented in this study is openly available on IDLab-PreDiCT Open Data (http://predict.idlab.ugent.be/open_data/) under the section **Context-aware lifestyle monitoring**. This dataset is licensed under CC BY-SA 4.0.

## Appendix B. Results of the General Models per CDS and MHEALTH for Each Participant

**Table A1:** Results of the general models per CDS and MHEALTH for participant 0.

Training Data	Balanced Accuracy	F1-Macro	F1-Micro	Logloss
CDS #1	0.63	0.29	0.86	0.58
CDS #2	0.71	0.34	0.90	0.40
CDS #3	0.77	0.34	0.72	0.57
CDS #4	0.80	0.32	0.69	0.55
CDS #5	0.80	0.32	0.63	0.60
CDS #6	0.80	0.33	0.71	0.55
CDS #7	0.77	0.37	0.52	0.80
CDS #8	0.76	0.30	0.54	0.80
MHEALTH	0.38	0.13	0.22	2.69

**Table A2:** Results of the general models per CDS and MHEALTH for participant 1.

Training Data	Balanced Accuracy	F1-Macro	F1-Micro	Logloss
CDS #1	0.79	0.54	0.81	0.58
CDS #2	0.81	0.57	0.89	0.35
CDS #3	0.83	0.56	0.81	0.42
CDS #4	0.81	0.56	0.80	0.44
CDS #5	0.82	0.56	0.81	0.38
CDS #6	0.85	0.56	0.80	0.46
CDS #7	0.83	0.57	0.83	0.32
CDS #8	0.82	0.56	0.81	0.40
MHEALTH	0.52	0.29	0.36	2.44

**Table A3:** Results of the general models per CDS and MHEALTH for participant 2.

Training Data	Balanced Accuracy	F1-Macro	F1-Micro	Logloss
CDS #1	0.85	0.36	0.82	0.56
CDS #2	0.89	0.37	0.87	0.39
CDS #3	0.84	0.36	0.82	0.44
CDS #4	0.84	0.44	0.80	0.50
CDS #5	0.83	0.44	0.80	0.51
CDS #6	0.81	0.43	0.76	0.59
CDS #7	0.85	0.45	0.83	0.41
CDS #8	0.86	0.45	0.83	0.37
MHEALTH	0.86	0.23	0.86	0.37

**Table A4:** Results of the general models per CDS and MHEALTH for participant 3.

Training Data	Balanced Accuracy	F1-Macro	F1-Micro	Logloss
CDS #1	0.81	0.49	0.76	0.70
CDS #2	0.82	0.52	0.80	0.49
CDS #3	0.80	0.51	0.76	0.56
CDS #4	0.80	0.50	0.74	0.59
CDS #5	0.81	0.52	0.76	0.51
CDS #6	0.80	0.52	0.77	0.51
CDS #7	0.83	0.53	0.81	0.43
CDS #8	0.81	0.52	0.78	0.46
MHEALTH	0.70	0.39	0.80	0.56

**Table A5:** Results of the general models per CDS and MHEALTH for participant 4.

Training Data	Balanced Accuracy	F1-Macro	F1-Micro	Logloss
CDS #1	0.50	0.41	0.68	0.98
CDS #2	0.52	0.42	0.74	0.79
CDS #3	0.50	0.43	0.67	0.78
CDS #4	0.49	0.41	0.59	0.90
CDS #5	0.49	0.41	0.63	0.83
CDS #6	0.48	0.42	0.63	0.76
CDS #7	0.49	0.42	0.66	0.70
CDS #8	0.51	0.42	0.68	0.69
MHEALTH	0.30	0.25	0.49	1.83

**Table A6:** Results of the general models per CDS and MHEALTH for participant 5.

Training Data	Balanced Accuracy	F1-Macro	F1-Micro	Logloss
CDS #1	0.77	0.33	0.64	0.99
CDS #2	0.81	0.43	0.77	0.60
CDS #3	0.79	0.51	0.70	0.66
CDS #4	0.78	0.46	0.64	0.75
CDS #5	0.78	0.51	0.66	0.67
CDS #6	0.78	0.43	0.68	0.65
CDS #7	0.78	0.55	0.69	0.62
CDS #8	0.78	0.45	0.67	0.61
MHEALTH	0.39	0.13	0.26	3.53

**Table A7:** Results of the general models per CDS and MHEALTH for participant 6.

Training Data	Balanced Accuracy	F1-Macro	F1-Micro	Logloss
CDS #1	0.75	0.31	0.67	0.70
CDS #2	0.82	0.39	0.79	0.46
CDS #3	0.76	0.35	0.63	0.65
CDS #4	0.78	0.36	0.68	0.60
CDS #5	0.78	0.36	0.67	0.61
CDS #6	0.78	0.36	0.64	0.73
CDS #7	0.79	0.45	0.66	0.61
CDS #8	0.79	0.36	0.66	0.61
MHEALTH	0.15	0.11	0.10	4.55

**Table A8:** Results of the general models per CDS and MHEALTH for participant 7.

Training Data	Balanced Accuracy	F1-Macro	F1-Micro	Logloss
CDS #1	0.73	0.48	0.88	0.44
CDS #2	0.63	0.44	0.59	0.81
CDS #3	0.55	0.37	0.35	1.10
CDS #4	0.62	0.41	0.51	0.91
CDS #5	0.54	0.38	0.33	1.05
CDS #6	0.54	0.36	0.34	1.11
CDS #7	0.52	0.31	0.28	1.20
CDS #8	0.58	0.38	0.41	1.02
MHEALTH	0.39	0.12	0.21	3.87

**Table A9:** Results of the general models per CDS and MHEALTH for participant 8.

Training Data	Balanced Accuracy	F1-Macro	F1-Micro	Logloss
CDS #1	0.84	0.36	0.80	0.62
CDS #2	0.80	0.35	0.84	0.44
CDS #3	0.83	0.36	0.81	0.47
CDS #4	0.81	0.36	0.77	0.53
CDS #5	0.82	0.36	0.80	0.44
CDS #6	0.77	0.43	0.79	0.49
CDS #7	0.83	0.45	0.86	0.41
CDS #8	0.79	0.44	0.81	0.40
MHEALTH	0.88	0.31	0.88	0.51

**Table A10:** Results of the general models per CDS and MHEALTH for participant 9.

Training Data	Balanced Accuracy	F1-Macro	F1-Micro	Logloss
CDS #1	0.90	0.56	0.94	0.23
CDS #2	0.91	0.56	0.95	0.20
CDS #3	0.92	0.57	0.95	0.21
CDS #4	0.92	0.57	0.94	0.21
CDS #5	0.91	0.57	0.94	0.22
CDS #6	0.93	0.57	0.95	0.21
CDS #7	0.92	0.57	0.95	0.21
CDS #8	0.92	0.57	0.94	0.21
MHEALTH	0.81	0.60	0.90	0.29

**Table A11:** Results of the general models per CDS and MHEALTH for participant 10.

Training Data	Balanced Accuracy	F1-Macro	F1-Micro	Logloss
CDS #1	0.90	0.53	0.90	0.43
CDS #2	0.93	0.55	0.93	0.39
CDS #3	0.92	0.55	0.92	0.33
CDS #4	0.93	0.55	0.93	0.32
CDS #5	0.92	0.54	0.92	0.31
CDS #6	0.92	0.54	0.92	0.35
CDS #7	0.92	0.55	0.92	0.31
CDS #8	0.92	0.54	0.92	0.34
MHEALTH	0.91	0.67	0.92	0.37

**Table A12:** Results of the general models per CDS and MHEALTH for participant 11.

Training Data	Balanced Accuracy	F1-Macro	F1-Micro	Logloss
CDS #1	0.79	0.48	0.93	0.30
CDS #2	0.76	0.47	0.92	0.26
CDS #3	0.93	0.50	0.95	0.19
CDS #4	0.94	0.51	0.95	0.18
CDS #5	0.92	0.51	0.95	0.23
CDS #6	0.91	0.63	0.95	0.20
CDS #7	0.81	0.60	0.94	0.21
CDS #8	0.86	0.49	0.94	0.22
MHEALTH	0.89	0.49	0.84	0.70

**Table A13:** Results of the general models per CDS and MHEALTH for participant 12.

Training Data	Balanced Accuracy	F1-Macro	F1-Micro	Logloss
CDS #1	0.96	0.49	0.96	0.22
CDS #2	0.91	0.47	0.91	0.29
CDS #3	0.93	0.38	0.93	0.25
CDS #4	0.92	0.47	0.92	0.27
CDS #5	0.92	0.47	0.92	0.30
CDS #6	0.90	0.47	0.91	0.31
CDS #7	0.93	0.48	0.93	0.26
CDS #8	0.90	0.47	0.90	0.32
MHEALTH	0.70	0.39	0.70	1.70

**Table A14:** Results of the general models per CDS and MHEALTH for participant 13.

Training Data	Balanced Accuracy	F1-Macro	F1-Micro	Logloss
CDS #1	0.83	0.69	0.87	0.63
CDS #2	0.72	0.58	0.79	0.54
CDS #3	0.75	0.66	0.72	0.69
CDS #4	0.69	0.62	0.76	0.63
CDS #5	0.74	0.66	0.80	0.47
CDS #6	0.72	0.65	0.78	0.55
CDS #7	0.69	0.63	0.77	0.60
CDS #8	0.70	0.63	0.78	0.55
MHEALTH	0.66	0.44	0.68	0.98

**Table A15:** Results of the general models per CDS and MHEALTH for participant 14.

Training Data	Balanced Accuracy	F1-Macro	F1-Micro	Logloss
CDS #1	0.53	0.55	0.66	1.87
CDS #2	0.74	0.75	0.89	0.51
CDS #3	0.76	0.76	0.89	0.38
CDS #4	0.77	0.77	0.89	0.32
CDS #5	0.85	0.80	0.91	0.25
CDS #6	0.94	0.83	0.93	0.25
CDS #7	0.92	0.83	0.93	0.20
CDS #8	0.93	0.83	0.92	0.23
MHEALTH	0.93	0.83	0.93	0.24

**Table A16:** Results of the general models per CDS and MHEALTH for participant 15.

Training Data	Balanced Accuracy	F1-Macro	F1-Micro	Logloss
CDS #1	0.93	0.64	0.93	0.21
CDS #2	0.99	0.66	0.99	0.10
CDS #3	1.00	1.00	1.00	0.03
CDS #4	1.00	1.00	1.00	0.03
CDS #5	1.00	1.00	1.00	0.02
CDS #6	1.00	1.00	1.00	0.03
CDS #7	1.00	1.00	1.00	0.02
CDS #8	1.00	1.00	1.00	0.05
MHEALTH	0.73	0.41	0.76	0.42

**Table A17:** Results of the general models per CDS and MHEALTH for participant 16.

Training Data	Balanced Accuracy	F1-Macro	F1-Micro	Logloss
CDS #1	0.76	0.34	0.88	0.53
CDS #2	0.72	0.30	0.55	0.85
CDS #3	0.73	0.32	0.63	0.61
CDS #4	0.74	0.42	0.73	0.56
CDS #5	0.72	0.39	0.62	0.69
CDS #6	0.72	0.39	0.61	0.69
CDS #7	0.70	0.40	0.66	0.60
CDS #8	0.71	0.40	0.64	0.64
MHEALTH	0.23	0.07	0.07	4.02

**Table A18:** Results of the general models per CDS and MHEALTH for participant 17.

Training Data	Balanced Accuracy	F1-Macro	F1-Micro	Logloss
CDS #1	0.82	0.59	0.84	0.56
CDS #2	0.80	0.57	0.89	0.45
CDS #3	0.76	0.53	0.81	0.51
CDS #4	0.76	0.42	0.75	0.64
CDS #5	0.79	0.45	0.76	0.66
CDS #6	0.80	0.58	0.86	0.45
CDS #7	0.77	0.54	0.81	0.45
CDS #8	0.78	0.57	0.85	0.42
MHEALTH	0.77	0.39	0.66	0.74
